# Mode of action of silver-based perovskite against Gram-negative bacteria

**DOI:** 10.1128/spectrum.01648-24

**Published:** 2024-12-10

**Authors:** Fereshteh Fani, Cyrus Talebpour, Philippe Leprohon, Hossein Salimnia, Houshang Alamdari, Marc Ouellette

**Affiliations:** 1 Département de Microbiologie, Infectiologie et Immunologie, Faculté de Médecine, Centre de recherche en infectiologie du Centre de Recherche CHU de Québec, Université Laval, Québec, Canada; 2 Department of Mining, Metallurgical and Materials Engineering, Université Laval, Québec, Canada; 3 Department of Pathology, School of Medicine, Children’s Hospital of Michigan, Wayne State University, Detroit Medical Center, Detroit, Michigan, USA; University of Saskatchewan, Saskatoon, Saskatchewan, Canada

**Keywords:** silver, perovskite, reactive oxygen species, lipid peroxidation, efflux pump, resistance, Gram-negative bacteria

## Abstract

Although silver is known for its antibacterial activity, its exact mode of action remains unclear. In our previous work, we described AgNbO_3_ nanoparticles (AgNbO_3_ NPs) prepared using a ceramic method, followed by high-energy and low-energy ball-milling processes, which exhibited antimicrobial activity with negligible release of Ag^+^ in deionized water. Here, we investigated thoroughly the mode of action of these AgNbO_3_ NPs against *Escherichia coli*. Drastic morphological changes in *E. coli* were observed after their exposure to AgNbO_3_ NPs. In addition to cellular damage, AgNbO_3_ NPs induced the production of reactive oxygen species and lipid peroxidation, likely following the release of small amounts of Ag^+^. This was concluded from the characterization of mutants resistant to AgNbO_3_ NPs that showed cross-resistance to AgNO_3_, impaired reactive oxygen species production and lipid peroxidation, and harbored a key mutation in a two-component regulatory system regulating an Ag^+^ efflux pump. We calculated, however, that the quantity of Ag^+^ released from AgNbO_3_ NPs is not sufficient by itself to lead to bacterial death. We propose that bacterial contact with the AgNbO_3_ NPs in combination with Ag^+^ release is necessary for the mode of action of AgNbO_3_ NPs.

**IMPORTANCE:**

Silver is known for its antibacterial activity, but its exact mode of action remains unclear. Here, we investigated thoroughly the mode of action of AgNbO_3_ nanoparticles against *Escherichia coli*. Our data suggest that AgNbO_3_ nanoparticles have dual effects on the cell and that both are required for its lethal action.

## INTRODUCTION

Silver has been used as an antimicrobial since antiquity, yet its mechanism of action is still ill-defined. It is unlikely that Ag^+^ has a specific cellular target, but the binding of Ag^+^ to various cellular components is intimately associated with membrane disruption and the production of reactive oxygen species (ROS) (1–3). These processes are thought to be key determinants of the mode of action of silver ions in clinically important Gram-positive and Gram-negative bacteria (1, 4–8). The continuous increase of antimicrobial resistance has called for alternatives to antimicrobials, and considerable efforts are devoted to the development of silver nanoparticles as alternatives to antimicrobials and disinfectant compounds. Indeed, silver nanoparticles are effective and importantly have a broad spectrum of antimicrobial activity against bacterial and fungal species (9). It is thought that the antibacterial activity is related to the Ag^+^ ions that are released from the nanoparticles (1, 10, 11). Silver nanoparticles are now used as antimicrobials in a wide range of medical applications such as wound dressings, fabrics, medical devices, topical creams and ointments, water purification, agriculture, antimicrobial coatings of surfaces, as well as in personal care products (8, 9, 12). However, the increasing use of silver nanoparticles in medical and non-medical applications carries a risk of selecting microorganisms resistant to silver. Indeed, silver-resistant bacteria have now been described (13), and the efflux of Ag^+^ seems to be a prominent mechanism of resistance. In many species, this efflux is mediated by the nine genes containing *sil* operon, encoding a multiprotein efflux pump and regulators (7, 14, 15).

We described previously AgNbO_3_ nanoparticles (AgNbO_3_ NPs) prepared by a ceramic method followed by high-energy and low-energy ball-milling processes (16). These AgNbO_3_ NPs demonstrated antimicrobial activity but did not release a significant amount of Ag^+^ ions (much less than the minimum inhibition concentration determined for Ag^+^ ions) when incubated for up to a month in either deionized water or acidic HCl solution (pH 4.0) (16). Since the antimicrobial activity of silver nanoparticles is thought to be associated with Ag^+^ release, it is possible that the antimicrobial activity of AgNbO_3_ NPs is mediated differently. Here, we report on the study of this putative distinctive mode of killing for AgNbO_3_ NPs by investigating thoroughly their mode of action against *Escherichia coli*.

## MATERIALS AND METHODS

### Chemicals

Silver-based perovskites (AgNbO_3_ nanoparticles) were synthesized using the ceramic method as described previously (16), and in this study are referred to as AgNbO_3_ NPs. This product was used in two forms including AgNbO_3_ powders (AgNbO_3_ NPs) and as a mixture with polymethyl methacrylate polymers (PMMA) (Surgical simplex P radiopaque powder, Stryker) in the form of discs (AgNbO_3_-PMMA) (17). Briefly, to make 4% AgNbO_3_ or Ag_2_O discs, powders from either AgNbO_3_ or Ag_2_O NPs were mixed with PMMA cement powder in a desired wt/wt ratio. Then liquid monomer (Simplex P liquid, Stryker) was added to the mixture and mixed until a homogenous paste was formed. This was immediately cast in a silicon mold with an inner diameter of 12 mm and depth of 3 mm. After solidification, the discs were released from the mold and used in subsequent experiments. Ionic silver in the form of silver nitrate salt (AgNO_3_) was also included in some experiments for comparison with AgNbO_3_.

### Bacterial strains, culture conditions

The three bacterial strains used in this study are *Klebsiella pneumoniae* CCRI-566, *Escherichia coli* CCRI-503, and *E. coli* CCRI-21520. The latter contains the *sil* operon and was used for generating resistant mutants. All strains were grown in Luria Bertani (LB) broth at 37°C under agitation at 200 rpm. Two independent clones of each *E. coli* strain were used in an attempt to generate cells resistant to AgNbO_3_ NPs and AgNO_3_ by stepwise concentration increments in LB broth medium.

### Growth curve and drug susceptibility

The growth of bacterial cells was determined using an automated incubation system (Biospa, BioTek) integrated to a Cytation 5 multimode reader (BioTek). Ten microliters of 1  ×  10^7^ CFU/mL was inoculated in 990 µL of LB medium in the absence or presence of AgNbO_3_ NPs at concentrations of 8, 16, and 32 µg/mL in a Falcon 24-well plate. The plate was incubated at 37°C, and the optical density was read at a wavelength of 600 nm (OD_600_) at 1 h intervals after shaking for 10  s using the Cytation 5 multimode reader. Minimum inhibitory concentration (MIC) assays were performed by a broth microdilution method in 96-well plates according to recommendations from CLSI. All MICs were determined from at least three independent biological cultures.

### Evaluating the antimicrobial effect of AgNbO_3_ discs on *E. coli*

AgNbO_3_ discs were immersed for 2 h in 1 mL of an overnight *E. coli* CCRI-503 culture grown in LB broth. Discs were then air-dried under sterile conditions for 20 min. The presence of live bacteria on the discs was evaluated by transferring these into 1 mL of fresh LB medium which was then incubated at 37°C for a duration of 20 h with OD_600_ measurements at 1 h intervals. To mitigate the variation in antimicrobial effects of discs, we tested three independent AgNbO_3_ discs with at least four biological replicates.

### Transmission electron microscopy

Transmission electron microscopy (TEM) was used to examine *E. coli* treated with AgNbO_3_ discs and NPs. A 10 mL overnight culture of *E. coli* was incubated with AgNbO_3_ NPs for 2 h, centrifuged, and the pellets were fixed in 1 mL of 2.5% glutaraldehyde in 0.1 M sodium cacodylate buffer pH 7.3 at 4°C. In the case of AgNbO_3_ or Ag_2_O discs, after 2 h contact with an overnight culture of *E. coli*, the discs were transferred to a new tube containing 10 mL of LB medium. The *E. coli* cells were detached from the disc’s surface by vortexing, centrifuged, and fixed as described above. After being washed with cacodylate buffer, cells were embedded in low melting point agarose and dehydrated repeatedly with increased concentrations of ethanol. The fixed cells were embedded in epoxy resin and sliced into an approximately 80 nm with a diamond knife. The specimen was placed onto a small copper grid and was further positively stained with uranyl acetate. TEM analysis was performed with a JEM-1230 electron microscope (JEOL, Japan) at the imaging and microscopy platform of the Institute of Integrative Biology and Systems of Université Laval.

### Detection of reactive oxygen species

The intracellular ROS accumulation was measured using cell permeant 2′,7′-dichlorodihydrofluorescein diacetate (H2DCFDA) dye (Invitrogen by Thermo Fisher Scientific, Canada). Briefly, overnight bacterial cultures were diluted 1:250 in 100 mL LB in a 500 mL flask. The cultures were grown to an OD_600_ of 0.1, then silver perovskite or hydrogen peroxide (H_2_O_2_) was added at a final concentration of 16 µg/mL and 1 mM, respectively. For hydroxyl radicals quenching, thiourea (Sigma Aldrich) was added to bacterial culture at a final concentration of 150 mM at the same time as silver perovskite or H_2_O_2_. One milliliter aliquots was collected after 15 min upon the additions of the analytes, centrifuged, and washed once with phosphate buffered saline (PBS) at pH 7.2. The DCFDA dye was added to a final concentration of 10 µM and incubated for 1 h at 37°C in the dark. Cells were washed once and resuspended in 500 µL of PBS. The fluorescence signal of a 200 µL aliquot was analyzed using a Victor fluorometer (Perkin-Elmer, Waltham, MA, USA) at 485 nm excitation and 535 nm emission wavelengths. Results are expressed as relative fluorescence units and were normalized according to the number of live cells. A minimum of three independent experiments have been performed for each condition.

### Lipid peroxidation assay

Malondialdehyde (MDA), a natural biomarker of lipid peroxidation, was measured using an OxiSelect thiobarbituric acid-reactive substances (TBARS) assay kit (STA-330) (Cell Biolabs Inc., San Diego, CA, USA) according to the manufacturer’s recommendations. Briefly, an overnight bacterial culture was diluted 250-fold in fresh LB medium and grown aerobically at 37°C with agitation to the early log phase (OD_600_ = 0.12). The 50 mL bacterial cultures were treated with AgNbO_3_ nanoparticles (32 µg/mL, i.e., 2× the MIC) with and without thiourea for 1 h before samples of treated and untreated controls were analyzed using the TBARS kit. Kanamycin (32 µg/mL, i.e., 2× the MIC), an aminoglycoside antibiotic known to initiate oxidative stress *in vivo* (18), was used as a positive control. The MDA-TBA formed from the reaction of MDA in samples with TBARS was measured by reading the absorbance at 532 nm. The level of MDA was determined using the MDA standard curve and expressed as micro-molar per 10^9^ cells analyzed.

### Expression of *sil*C by qRT-PCR

Total RNA was extracted using the RNeasy plus mini kit (Qiagen), according to the manufacturer’s instructions. Reverse transcription was performed using the Superscript II reverse transcriptase (Invitrogen), followed by RNA degradation with RNase H (Invitrogen). Primers for detection of *sil*C expression were designed using Primer3web version 4.1.0. Quantitative PCR was performed using SYBR Green Supermix (BioRad) and a Rotor Gene-3000 (Corbett Research), with an initial denaturation at 95°C for 4 min followed by 40 cycles consisting of denaturation at 94°C for 20 s, annealing at 60°C for 20 s, and extension at 72°C for 20 s. All cycle threshold (Ct) values were derived from the average of three technical replicates for each biological replicate. The amount of PCR products was determined using the relative standard curve method. The standard growth curve parameters were R2 ≥ 0.98, and amplification efficiency ≥ 90%. Normalization was done with the 16S rRNA gene. The results were obtained from three biological replicates. Statistical analysis was performed using a two-tailed Student’s *t*-test.

### Whole genome sequencing and analysis

Genomic DNA (gDNA) was extracted from *E. coli* (CCRI-21520) wild-type, AgNO_3_-resistant, and AgNbO_3_-resistant clones (called as Ag64 and Nb64, respectively) using the Wizard genomic DNA purification kit (Promega). Illumina DNA Prep sequencing libraries were prepared from gDNA according to the manufacturer’s instructions. The size distribution of the libraries was validated using a 2100 Bioanalyzer and high-sensitivity DNA chips (Agilent Technologies). Paired-end sequencing was performed on an Illumina NovaSeq6000. The genome of the wild-type sample was assembled from the sequencing reads using Spades (version 3.15.1) (19), and the contigs were annotated using RAST (20, 21). Sequence reads from the resistant mutants were aligned to the wild-type genome assembly using bwa-mem (22). The maximum number of mismatches was 4, the seed length was 32, and two mismatches were allowed within the seed. Read duplicates were marked using Picard, and we applied GATK (23, 24) for single-nucleotide polymorphism and indels discovery. The sequence reads for *Escherichia coli* CCRI-21250 have been deposited in the Sequence Reads Archive ( https://www.ncbi.nlm.nih.gov/bioproject/1022057) under sample accession SAMN37586940, while the accession numbers SAMN37586934, SAMN37586935, SAMN37586938, and SAMN37586939 have been assigned to Ag64.1, Ag64.2, Nb64.1, and Nb64.2, respectively.

### Silver release measured by Microwave Plasma Atomic Emission Spectrometry

The release of silver ions from AgNbO_3_ NPs was evaluated in deionized water and LB medium alone and in the presence of bacteria. Briefly, two 5 mL of the AgNbO_3_ NPs were prepared at concentrations of 8, 16, and 32 µg/mL in deionized water and LB medium. After adding 10 µL of overnight culture of *E. coli* CCRI-503 to one tube of each concentration, the tubes were incubated at 37°C incubator with 200 rpm shaker. After 24 h, all tubes were taken from the incubator and centrifuged for 10 min at 13,000 rpm, then the supernatants were filtered (filter pore size 0.22 µM). A 3 mL aliquot of each sample was sent for Microwave Plasma Atomic Emission Spectrometry (MP-AES) measurement, and 1 mL was used to determine its antibacterial activity against *E. coli*. The obtained values of the MP-AES measurement were based on a calibration curve made with an Ag^+^ equivalent to 0.04–10 ppm Ag from a certified standard solution of 1,000 ppm Ag and centered at the Ag emission value of 328 nm. As an internal control, we used the AgNO_3_ solutions at concentrations of 4, 8, 16, and 32 µg/mL in deionized water and LB.

## RESULTS AND DISCUSSION

The use of silver nanoparticles as an antimicrobial agent has been extensively documented, and numerous investigations have been carried out to reveal their antibacterial mechanisms. The antimicrobial activity of silver nanoparticles is attributed to the release of silver ions which interact with various cellular components resulting in membrane damage, the production of ROS, and eventually to the death of bacteria but also of fungi (2, 3, 10, 25–29). However, other studies have suggested that microbial contact with silver nanoparticles may also play a role in enhancing their antimicrobial effect (30, 31).

**Fig 1:**
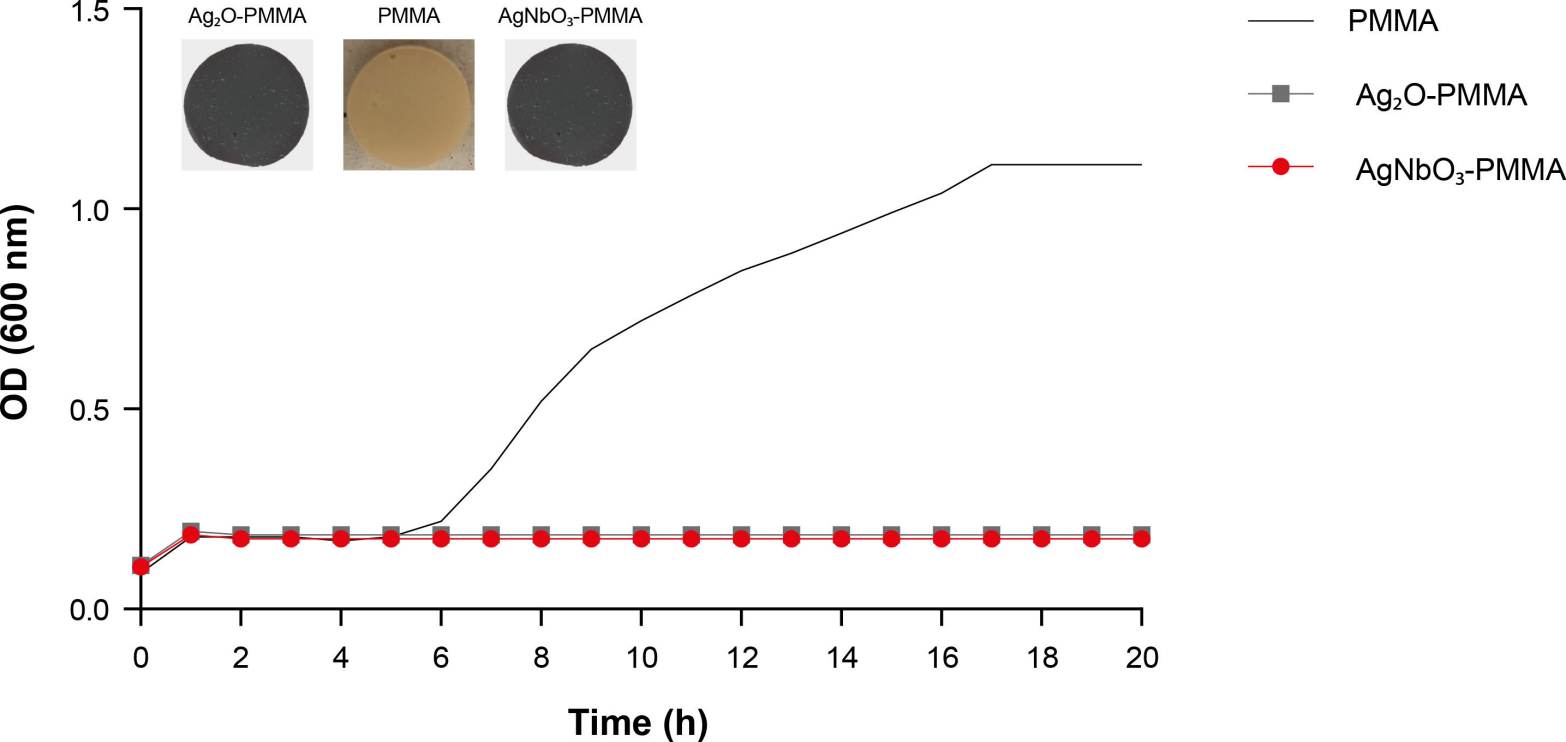
Antimicrobial activity of AgNbO_3_-PMMA discs. The PMMA discs are shown in the inset. One milliliter of an overnight culture of *E. coli* CCRI-503 was incubated for 2 h with PMMA, Ag_2_O-PMMA, or AgNbO_3_-PMMA discs. The discs were removed, gently air-dried, and incubated in LB medium. The *E. coli* growth was measured using a Cytation 5 multimode reader at OD (600 nm) with 1 h intervals.

**Fig 2:**
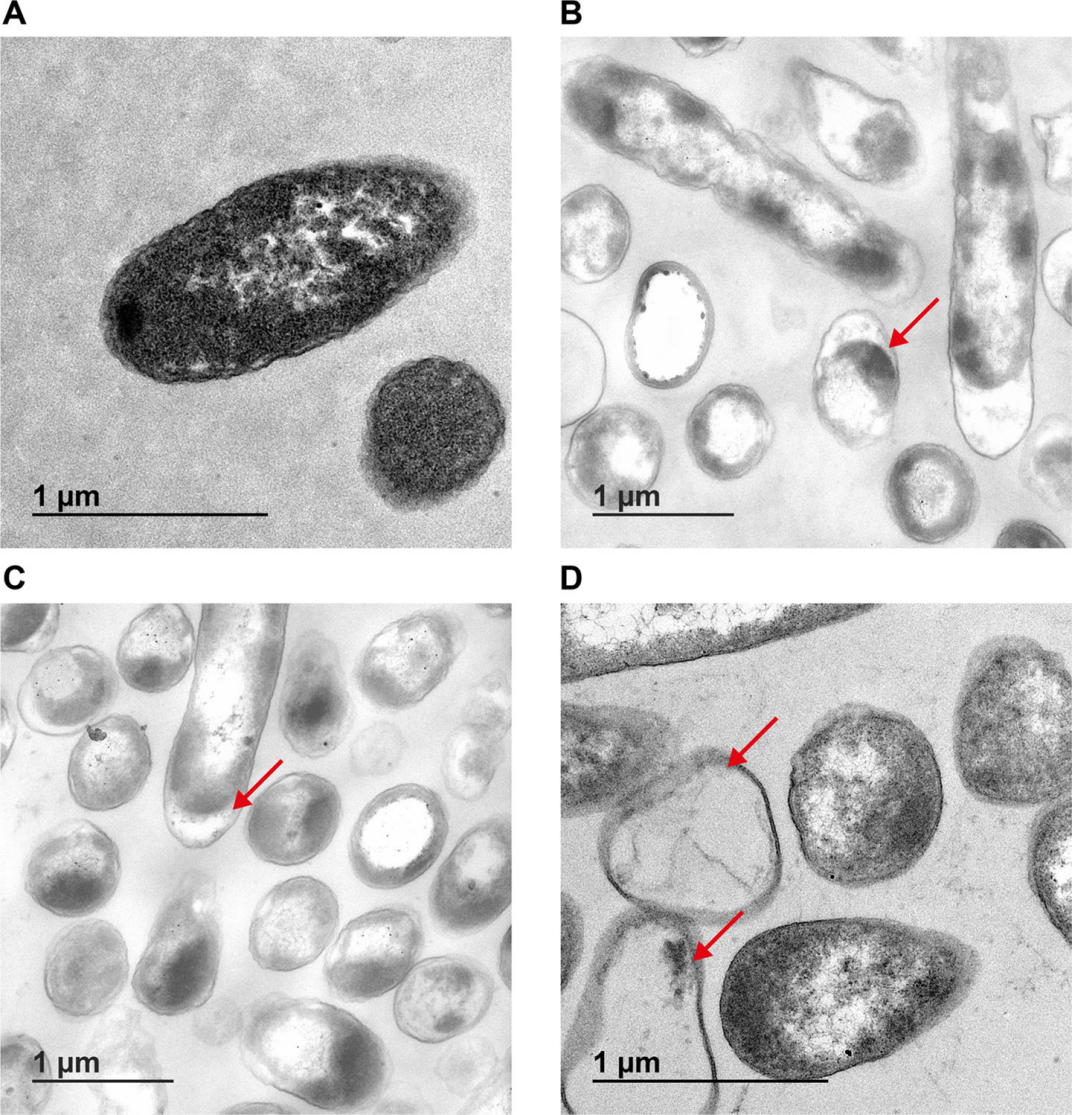
Transmission electron microscopy imaging of *E. coli* treated with AgNbO_3_. *E. coli* CCRI-503 was incubated with PMMA control discs (**A**), AgNbO_3_-PMMA discs (**B**), Ag_2_O-PMMA discs (**C**), or with 32 µg/mL of AgNbO_3_ nanoparticles suspension (**D**). Arrows point to ghost cells and/or membrane defects.

Interested in the application of silver nanoparticles for antimicrobial surface coatings, we incorporated AgNbO_3_ NPs in PMMA mixture and prepared solid discs (17). PMMA is widely used as bone cement in orthopedic surgery. Using the same method, we also prepared Ag_2_O-PMMA discs which, in contrast to AgNbO_3_, have previously been shown to release Ag^+^ ions (16). Discs made of PMMA alone were used as a negative control. Cultures of *E. coli* CCRI-503 were let in contact with AgNbO_3_-PMMA, Ag_2_O-PMMA, or PMMA discs for 2 h. The discs were removed, air-dried, transferred into fresh LB medium, and incubated for a duration of 20 h with OD_600_ readings at 1 h intervals. While bacterial growth was observed in the tubes from the PMMA-disc control culture, the AgNbO_3_-PMMA and Ag_2_O-PMMA discs demonstrated potent antibacterial activity by preventing the growth of *E. coli* (Fig. 1). Of note, we have shown previously that AgNbO_3_-PMMA discs exhibited no toxicity on THP1 cells, whereas Ag_2_O-PMMA caused a significant reduction in THP1 survival (17). Cell damage is often associated with silver nanoparticles as part of their mode of action (6, 26, 27). To assess whether this is also the case with our AgNbO_3_-PMMA discs, we examined the integrity of *E. coli* cells by transmission electron microscopy after their contact with AgNbO_3_-PMMA discs or with PMMA control discs. *E. coli* cells incubated with PMMA control discs showed intact morphology with evenly distributed cytoplasm (Fig. 2A). However, *E. coli* cells incubated with AgNbO_3_-PMMA discs for 2 h demonstrated significantly altered bacterial cell wall integrity and cell shape, with the cell membrane detaching from the cell wall and the release of cytoplasmic content leading to ghost cells (Fig. 2B). Similar data were observed when *E. coli* was incubated with Ag_2_O-PMMA discs (Fig. 2C).

PMMA discs, while effective at killing *E. coli*, limited the work that could be done in our investigation on the mode of action of AgNbO_3_ NPs, and as a proxy, we decided to pursue AgNbO_3_ NPs in suspension as these allowed to determine an MIC. Previously, we have shown that AgNbO_3_ NPs had activity against *Pseudomonas aeruginosa* and *Staphylococcus aureus* (16). We now extended this spectrum of activity by demonstrating that *E. coli* and *Klebsiella pneumoniae*, two critical pathogens for which drug development is urgently required (32), are also killed by AgNbO_3_ NPs with an MIC of 16 µg/mL (Table 1). AgNO_3_ exhibited higher activity against these strains, as evidenced by a lower MIC ranging from 4 to 8 µg/mL (Table 1). Such a difference in MICs was expected given that AgNbO_3_ NPs are poorly soluble and release fewer Ag^+^ ions, thus a higher concentration is required to achieve the same effect as with AgNO_3_. Similar to AgNbO_3_-PMMA discs, incubation of *E. coli* CCRI-503 with AgNbO_3_ NPs in suspension for 2 h led to drastic changes in cell shape and cell wall integrity and to the generation of ghost cells (Fig. 2D).

**TABLE 1:** Minimum inhibitory concentration of AgNbO_3_ and AgNO_3_ against *E. coli* and *Klebsiella pneumoniae*

Stain or mutants	MIC of AgNbO_3_ (µg/mL)^*a*^	MIC of AgNO_3_ (µg/mL)
*E. coli* CCRI-503	16	4–8
*E. coli* CCRI-21520	16	4–8
*K. pneumoniae* CCRI-566	16	2–4
Ag64.1^*b*^	>512	>512
Ag64.2^*b*^	>512	>512
Nb64.1^*b*^	>512	>512
Nb64.2^*b*^	>512	>512

^*a*^ All MICs are the average of at least three biological replicates.

^*b*^ Mutants of *E. coli* CCRI-21520 selected for resistance to 64 µg/mL of either AgNO_3_ (Ag) or AgNbO_3_ (Nb).

**Fig 3:**
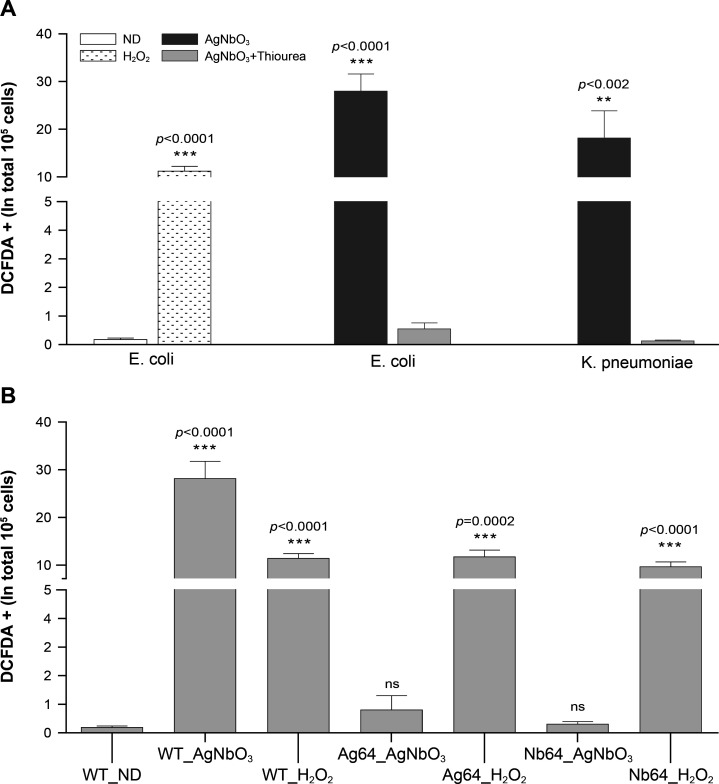
ROS induced by AgNbO_3_ nanoparticles. (A) ROS production was measured using DCFDA fluorescence of *E. coli* CCRI-503 following 15 min exposure to 1 mM H_2_O_2_, or of *E. coli* CCRI-503 and *K. pneumoniae* CCRI-566 following 15 min exposure to 16 µg/mL of AgNbO_3_ NPs with or without 150 mM of thiourea. Untreated *E. coli* (ND, no drug) cells were used as control. (B) ROS production of *E. coli* CCRI-21520 selected for resistance to silver derivatives. The DCFDA fluorescence signals of *E. coli* CCRI-21520 (WT), Ag64 (selected for resistance to AgNO_3_), or Nb64 (selected for resistance to AgNbO_3_) were measured following 15 min exposure to either 16 µg/mL of AgNbO_3_ or 1 mM H_2_O_2_. In all cases, the fluorescence signals were normalized to 10^6^ live cells. The results are the average of at least three independent experiments.

In addition to cellular damage, the production of ROS is the archetypal marker of the mode of action of Ag^+^ (10, 28, 33). As a positive control for ROS production, we used H_2_O_2_ (34) which indeed induced ROS in *E. coli* CCRI-503 (Fig. 3A) as measured with dichlorofluorescein diacetate (DCFDA). The same *E. coli* strain, when incubated with AgNbO_3_ NPs at the MIC (16 µg/mL) for 15 min, also showed a significant increase in ROS production which could be quenched by the addition of thiourea (Fig. 3A). Thiourea also rescued *E. coli* growth which was otherwise inhibited by AgNbO_3_ NPs (Fig. S1) and increased the MIC of AgNbO_3_ NPs to 128 µg/mL. AgNbO_3_ NPs, at 16 µg/mL, also had the capacity to induce ROS in *K. pneumoniae* (Fig. 3A). Consistent with the *E. coli* data, thiourea could prevent this ROS production (Fig. 3A).

**Fig 4:**
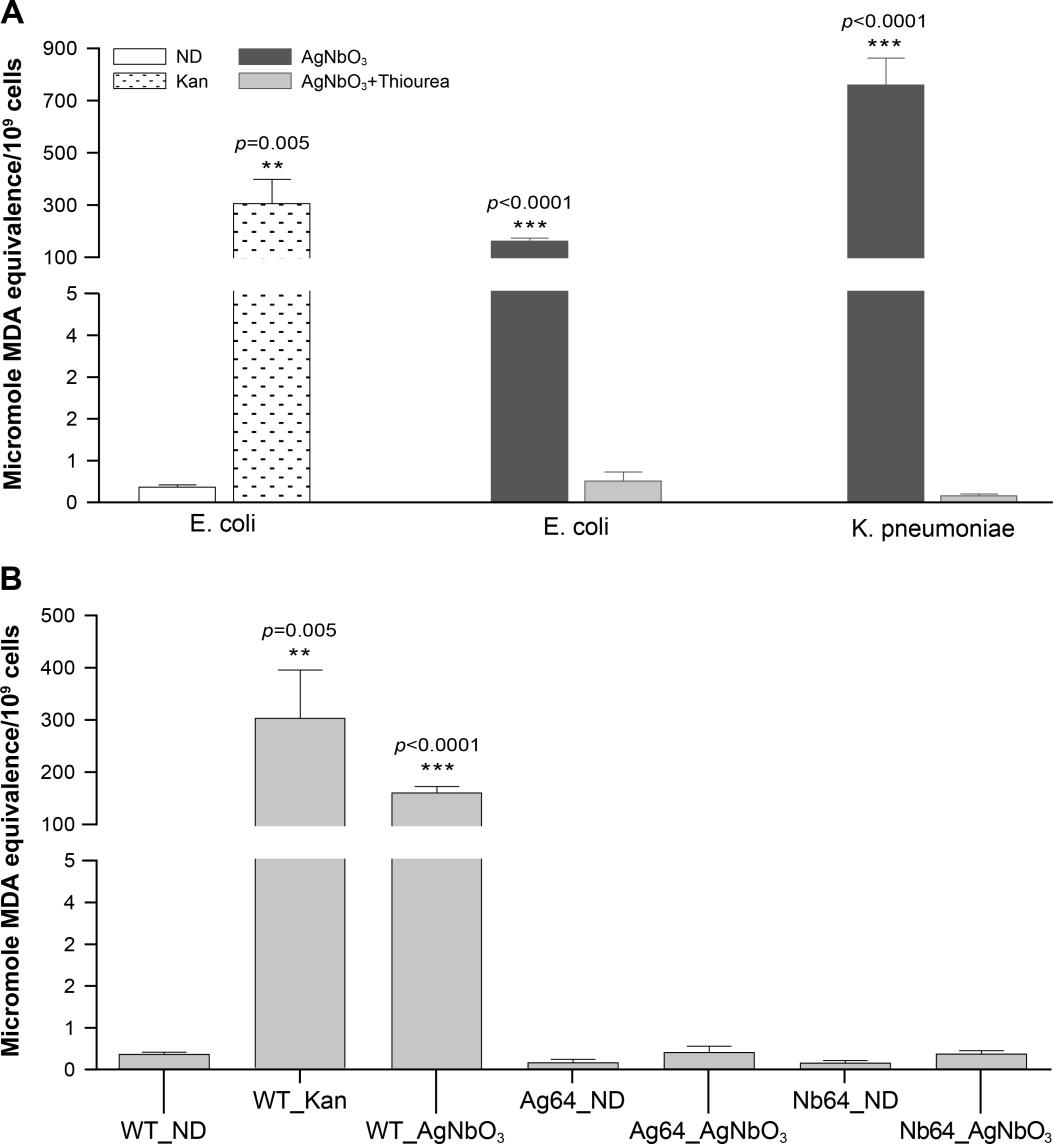
Lipid peroxidation measured in bacterial cells incubated with AgNbO_3_ nanoparticles. (A) The MDA level, a product of lipid peroxidation, was measured using the TBARS assay in *E. coli* CCRI-503 cells treated with 32 µg/mL of kanamycin (Kan), or in *E. coli* CCRI-503 and *K. pneumoniae* CCRI-566 treated with 32 µg/mL of AgNbO_3_ for 60 min with and without 150 µM of thiourea. Untreated *E. coli* (ND, no drug) cells were used as controls. (B) Lipid peroxidation in *E. coli* CCRI-21520 selected for resistance to silver derivatives. MDA level was measured using the TBARS assay in of *E. coli* CCRI- 21520 (WT) and mutants Ag64 (selected for resistance to AgNO_3_) and Nb64 (selected for resistance to AgNbO_3_) following 60 min exposure to 32 µg/mL of AgNbO_3_. In all cases, the MDA levels were normalized to 10^9^ live cells. The results are the average of at least three independent experiments.

The production of ROS is often associated with lipid peroxidation (35, 36). We investigated this effect by monitoring malondialdehyde (MDA) production, an end product of lipid peroxidation, using the TBARS assay (37). As a positive control, we used the antibiotic kanamycin (at 32 µg/mL), a known mediator of lipid peroxidation (18), which indeed induced lipid peroxidation in *E. coli* as determined by the TBARS assay (Fig. 4A). We also observed substantial lipid peroxidation when *E. coli* CCRI-503 or *K. pneumoniae* were exposed to 32 µg/mL of AgNbO_3_ NPs, which was alleviated after addition of thiourea (Fig. 4A).

**Fig 5:**
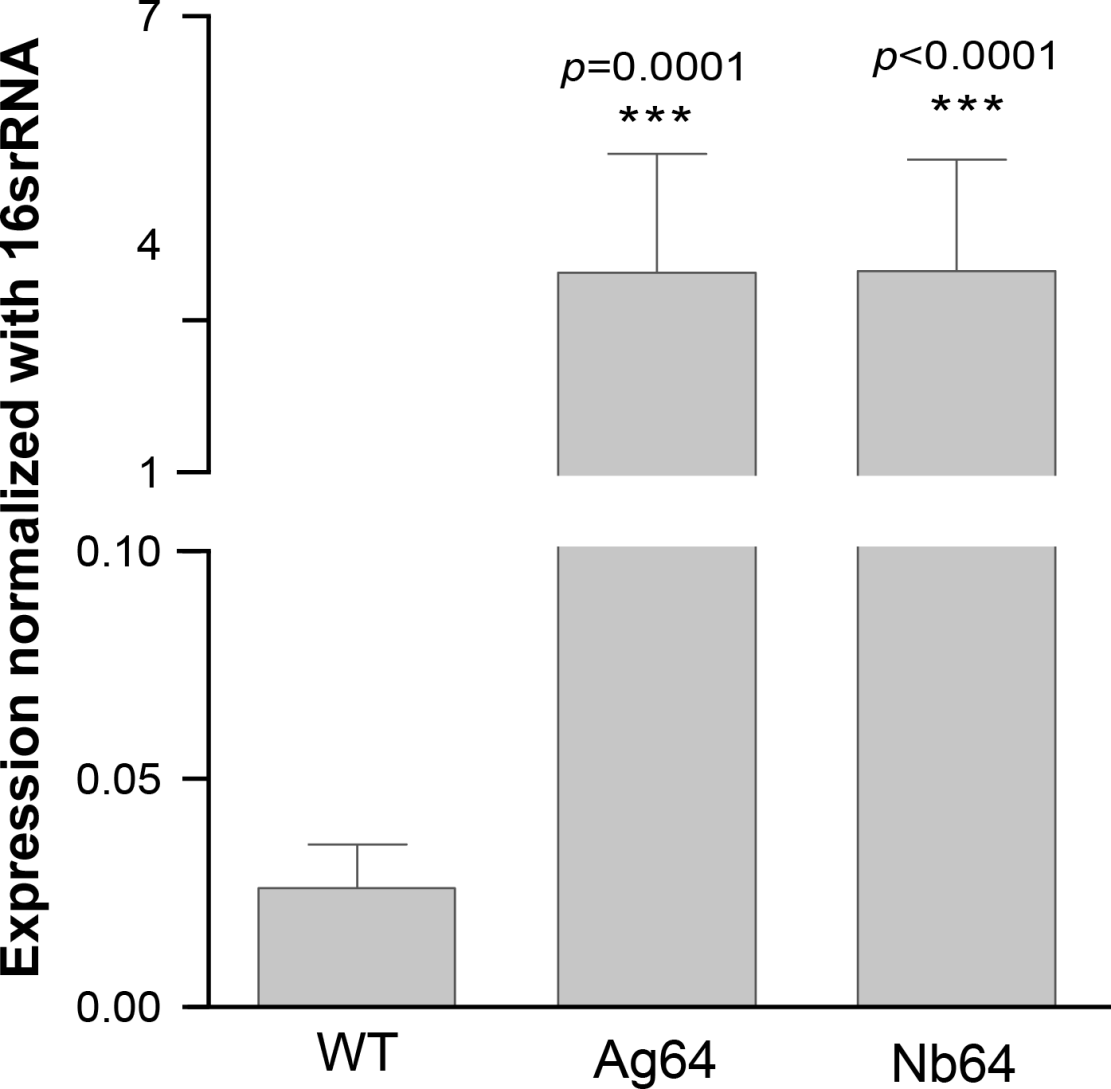
Expression of *sil*C in *E. coli* selected for resistance to silver containing compounds. Total RNAs were extracted from *E. coli* CCRI-21520 (WT), Ag64, and Nb64 (*E. coli* selected for resistance to AgNO_3_ and AgNbO_3_, respectively), converted to cDNA, and the expression of the *sil*C gene was determined by qRT-PCR. Ct values were calculated using standard curves of pooled cDNA. Results were normalized based on 16S rRNA expression. Results are the mean of three biological replicates.

Generating drug-resistant mutants and subsequently characterizing them through whole genome sequencing are helpful in understanding the mode of action of drugs (38, 39). We thus attempted to select *E. coli* CCRI-503 for resistance to AgNbO_3_ by incremental exposure to subtoxic concentrations of AgNbO_3_ NPs but this proved unsuccessful. This was not due to a specific property of AgNbO_3_ NPs as we also failed, using the same *E. coli* strain, in generating resistant mutants when using AgNO_3_. It has been described, however, that *E. coli* strains harboring the *sil* operon (5, 7, 40) have the potential to develop resistance to Ag^+^. We thus screened *E. coli* strains from our collection by PCR for three genes (*silR*, *silS*, and *silE*) part of the *sil* operon (Fig. S2A) using primers derived from the sequence of the plasmid PMG101 (Genbank accession AF067954). We obtained *silR* and *silE* PCR fragments of the expected size for a single strain (CCRI-21520) out of the 20 tested. We amplified the regions between *silR* and *silE* in CCRI-21520, and its sequencing revealed a *silS* orthologue but with a number of single nucleotide polymorphisms compared to the PMG101 sequence (Fig. S2B). The presence of the *sil* locus, however, was not associated *per se* with resistance as the MICs of AgNO_3_ and AgNbO_3_ NPs were similar for *E. coli* CCRI-21520 (*sil^+^*) and CCRI-503 (*sil^−^*) (Table 1). It was straightforward, however, to select for resistance against AgNO_3_ and AgNbO_3_ NPs from *E. coli* CCRI-21520 by stepwise selection up to 64 µg/mL. Two independent mutants were selected for both AgNO_3_ and AgNbO_3_ NPs, and these were designated as Ag64.1, Ag64.2 and Nb64.1, Nb64.2, respectively. Those mutants were highly resistant to the selective drug but also demonstrated cross-resistance; cells selected with AgNO_3_ were cross-resistant to AgNbO_3_ NPs and vice-versa (Table 1). We sequenced the genomes of all four mutants, and these were found to share most of their mutations (Table S1). Some mutations were in hypothetical proteins or in mobile element proteins (Table S1), but interestingly the *silS* gene had a G1337T transversion leading to a SilS S446I mutation in the four individual mutants (Table S1). SilS is a regulator of the SilCFBA efflux transporter (Fig. S2), and an S446I mutation was previously shown to lead to its constitutive activation (5, 15, 41). We thus tested the expression of *sil*C, a component of the Ag^+^ efflux pump, whose gene is under the control of SilS. An increase of at least 100-fold in the expression of *sil*C was observed in the Ag64 and Nb64 resistant mutants (Fig. 5). We noted that an aldehyde dehydrogenase was also mutated at the same position (V182A) in the four mutants (Table S1), but this was not further studied. For both resistant lines (Ag64 and Nb64), AgNbO_3_ NPs failed to induce ROS production. However, these cells were still capable of producing ROS when incubated with H_2_O_2_ (Fig. 3B). Similarly, treatment of the mutants with AgNbO_3_ NPs failed to lead to lipid peroxidation (Fig. 4B).

**Fig 6:**
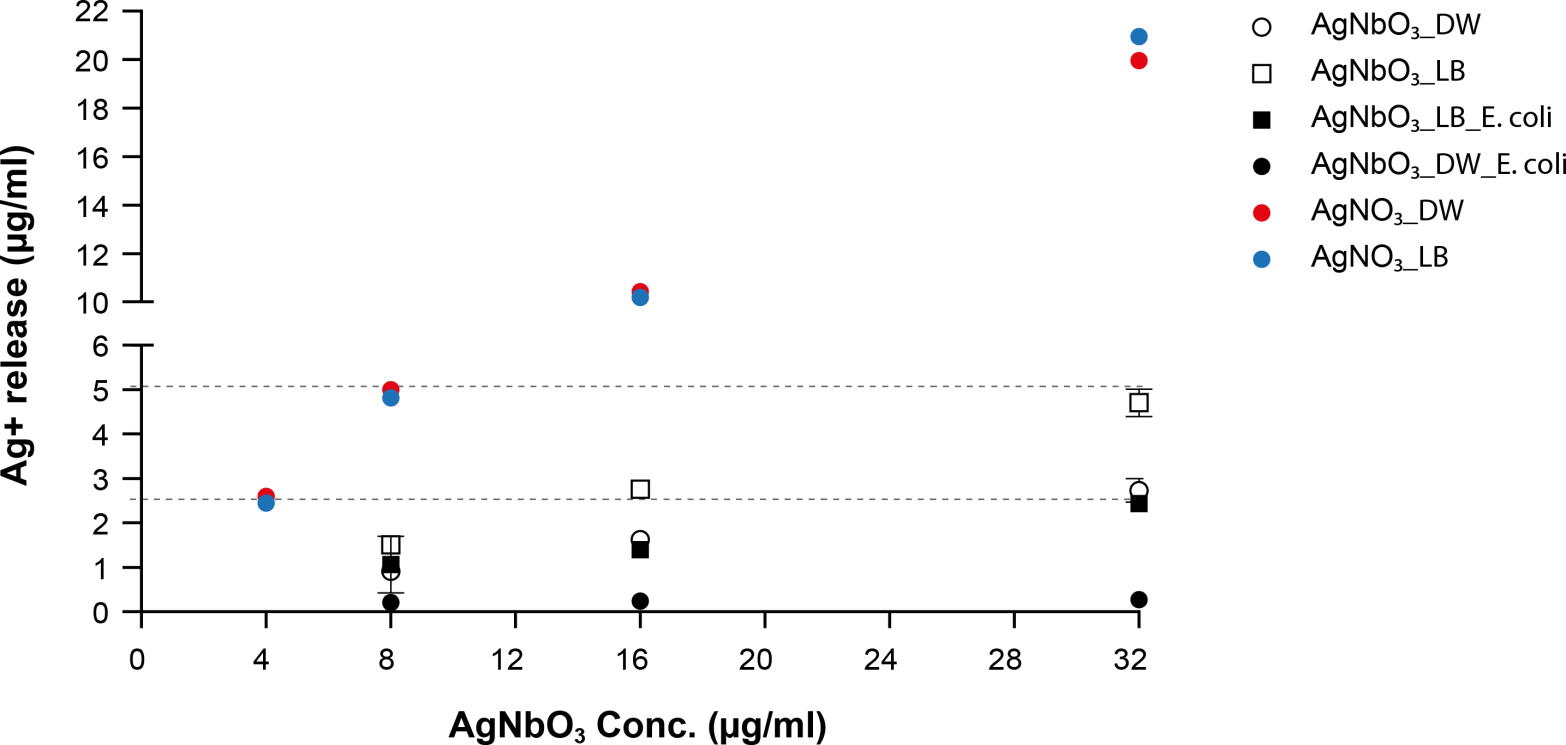
Silver (Ag^+^) release from AgNbO_3_ nanoparticles. The Ag^+^ level was measured by MP-AES. The AgNbO_3_ was incubated (i) in deionized water alone (DW) or with *E. coli* (DW_Ecoli); (ii) in LB medium alone (LB) or with *E. coli* (LB_Ecoli) for 24 h. Dashed lines represent Ag^+^ level (2.54 µg/mL and 5.08 µg/mL) equivalent to the MIC range of AgNO_3_ against *E. coli* (4.0–8.0 µg/mL) (Table 1). The level of Ag+ was also measured in AgNO_3_ solution in DW (red) or LB (blue). Results are the mean of three biological replicates.

While our data suggest that the mode of action of AgNbO_3_ NPs is mediated through the release of Ag^+^, we previously showed that there is minimal release of Ag^+^ from such NPs in aqueous solutions, at levels much lower than the MIC (16). To further explore this previous finding, we hypothesized that Ag^+^ release may vary in more complex medium or when bacteria are present. We thus incubated AgNbO_3_ NPs, or AgNO_3_ as a comparator, in deionized water and liquid LB medium in the presence or absence of *E. coli* and quantified the Ag^+^ released in the supernatant using MP-AES. As previously reported (16), negligible amount of Ag^+^ was released from AgNbO_3_ NPs in deionized water, irrespective of the presence of bacteria (Fig. 6). Although a higher concentration of Ag^+^ was detected in the supernatant of samples exposed to AgNbO_3_ NPs in LB medium compared to deionized water (Fig. 6), this difference is likely not due to an increased release of Ag^+^ in LB. Instead, it may be attributed to smaller-sized AgNbO_3_ NPs not fully settling after centrifugation in LB, causing MP-AES to detect these nanoparticles as Ag^+^. However, when the bacterial cells were present in the samples, these smaller nanoparticles could have been carried down with *E. coli*, resulting in an Ag^+^ release similar to that found in deionized water (Fig. 6). Of note, the level of Ag^+^ released from AgNO_3_ was higher in all the conditions tested (Fig. 6).

Importantly, the level of Ag^+^ released from AgNbO_3_ NPs in the LB medium was found to be below the equivalent of 5.08 µg/mL Ag^+^ (upper limit of MIC range) required to inhibit the growth of *E. coli* (Fig. 6; Table 1). To validate that the amount of Ag^+^ released from AgNbO_3_ NPs in LB medium is at a sub-inhibitory level for *E. coli*, we collected the supernatants of tubes incubated with 8, 16, and 32 µg/mL of AgNbO_3_ NPs in LB and tested their activity on *E. coli*. Interestingly, none of the supernatants were able to inhibit bacterial growth, confirming that the level of Ag^+^ released from AgNbO_3_ NPs was not sufficient to prevent *E. coli* growth. In contrast, the supernatant of AgNO_3_ in LB at concentrations ≥ 8 µg/mL completely inhibited *E. coli* growth, consistent with the inhibitory Ag^+^ levels detected by MP-AES (Fig. 6)

The majority of investigations on silver nanoparticles suggested that their antibacterial activity is attributed to the release of silver ions (1, 10, 11, 42). Our data indicate that AgNbO_3_ NPs inhibit *E. coli* growth through mechanisms leading to the accumulation of ROS and lipid peroxidation, which may be triggered by sub-MIC Ag^+^ release and by membrane disruption. The selection of mutants resistant to AgNbO_3_ NPs harboring a mutation in the *sil* locus also suggests a role for Ag^+^ release as part of their mode of action. However, the amount of Ag^+^ released is not sufficient to exert antibacterial activity, suggesting that a combination of cellular contact of the AgNbO_3_ NPs in addition to Ag^+^ release is necessary in initiating the events leading to bacterial death. Further investigation is warranted to elucidate how such putative contact contributes to the mode of action of AgNbO_3_ NPs.

## SUPPLEMENTAL MATERIAL

Supplemental material spectrum.01648-24-s0001.pdf Table S1; Fig. S1 and S2.

ASM does not own the copyrights to Supplemental Material that may be linked to, or accessed through, an article. The authors have granted ASM a non-exclusive, world-wide license to publish the Supplemental Material files. Please contact the corresponding author directly for reuse.
