# Supplementary material for: Mode of action of silver-based perovskite against Gram-negative bacteria
Source: Microbiol Spectr. 2024 Dec 10;13(1):e01648-24. doi: 10.1128/spectrum.01648-24 (PMC11705935; doi:10.1128/spectrum.01648-24)
Supplement: Supplemental material — Table S1; Fig. S1 and S2. [file spectrum.01648-24-s0001.pdf]

Table S1. Mutations following sequencing of $AgNbO_3$ and $AgNO_3$ resistant mutants

Mutated gene	Mutation (Nucleotide; amino acid changes)	AgNO3 or AgNbO3 Mutants			
		**Ag64.1**	**Ag64.2**	**Nb64.1**	**Nb64.2**
silS	G1337T; S446I	+	+	+	+
Predicted ATP-dependent endonuclease of the OLD family	1097-1098 Deletion; frameshift	+	+	-	-
	C1145T; R382H	+	+	-	-
	A991C; S331A	+	+	+	+
Mobile element protein	C160T; H54Y	+	+	-	+
	C200G; T67S	+	+	+	+
	T441 Insertion; frameshift	+	+	+	+
Aldehyde dehydrogenase (EC 1.2.1.3)	A545G; V182A	+	+	+	+
DNA integration/recombination/invertion protein	C91T; R31C	+	+	+	+
Mobile element protein	C5T; G2D	+	+	+	+
	C69A; R23S	+	+	+	+

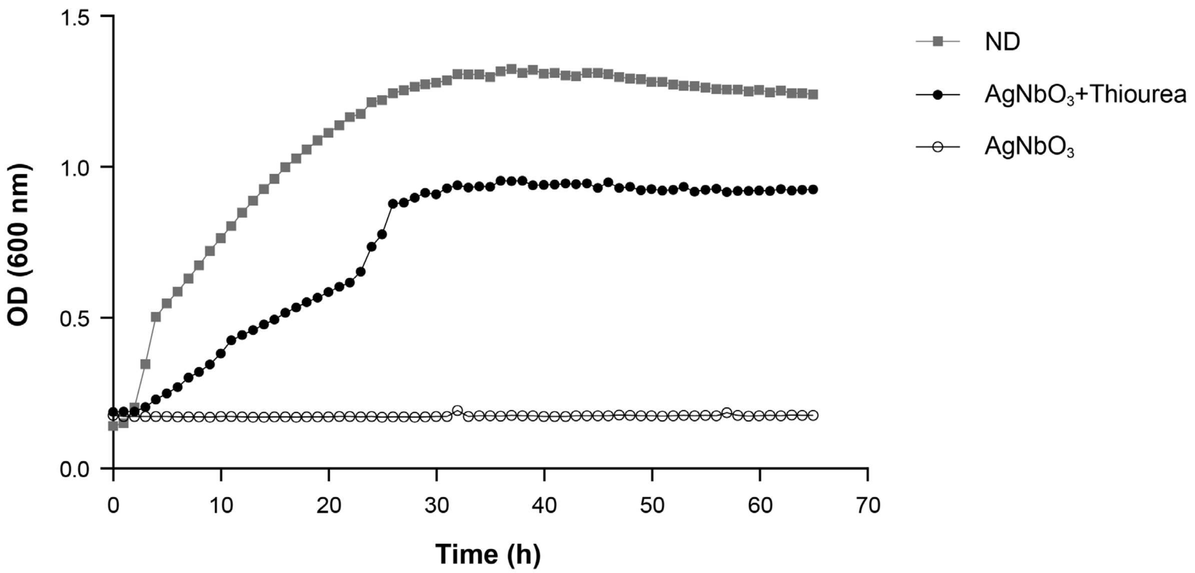

Figure S1. **Thiourea rescue the growth of *E. coli* CCRI-503 treated with $AgNbO_3$.**

Growth (OD 600) of *E. coli* CCRI-503 strain before (ND, no drug) and after exposure to 16 µg/ml of $AgNbO_3$ with and without 150 mM of thiourea was monitored at 1 h intervals for a duration of 60 h using a Cytation5 multimode reader equipped with a BioSpa robot and incubator.

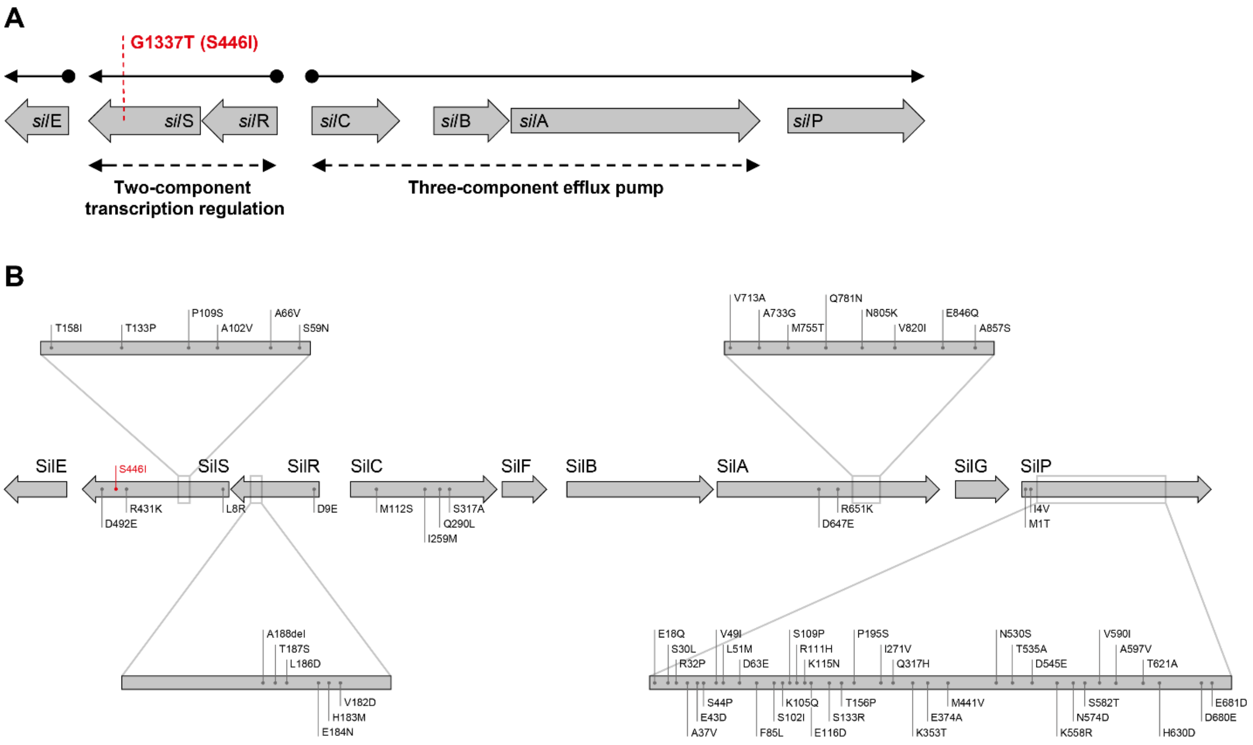

Figure S2. **Genetic architecture of the *sil* operon and coding differences between the *sil* operon of *E. coli* CCRI-21520 and PMG101**. A. The genetic architecture of the *sil* operon is shown. Grey arrows represent the *sil* operon genes. B. Sequencing of the *sil* operon from *E. coli* CCRI-21520 revealed numerous mutations compared with PMG101 sequence (gene bank accession number, AF067954). The position of each coding mutation is indicated by the number next to the arrows, with the first letter indicating the original amino acid from PMG101 and the second letter showing the change identified upon sequencing of *E. coli* CCRI-21520. The G1337T transversion leading to a SilS S446I mutation in both Ag64 and Nb64 (*E. coli* selected for resistance to $AgNO_3$ and $AgNbO_3$ respectively) is shown in red.
